# Rifaximin’s therapeutic spectrum: approved indications and experimental insights into emerging uses

**DOI:** 10.1007/s43440-026-00830-0

**Published:** 2026-02-23

**Authors:** Irene Palenca, Silvia Basili Franzin, Marcella Pesce, Giovanni Sarnelli, Giuseppe Esposito

**Affiliations:** 1 Neuropharmacology and Behavioral Science Laboratory, Santa Lucia Foundation (IRCCS Fondazione Santa Lucia), Rome, Italy; 2 Department of Physiology and Pharmacology “V. Erspamer”, Sapienza University of Rome, Piazzale Aldo Moro 5, 00185 Rome, Italy; 3 Department of Clinical Medicine and Surgery, ‘Federico II’ University of Naples, Via Pansini 5, Naples, Naples, Italy

**Keywords:** Rifaximin, Gut microbiota, Dysbiosis, Traveler’s diarrhea (TD), Irritable bowel syndrome with diarrhea (IBS-D), Hepatic encephalopathy (HE), Small intestinal bacterial overgrowth (SIBO), Pregnane x receptor (PXR), Inflammation, Gut-liver axis, Gut-brain axis

## Abstract

Since its 1987 approval, rifaximin has emerged as a gut-targeted, minimally absorbed antibiotic achieving high intestinal levels. Rifaximin works by blocking bacterial RNA polymerase to stop bacterial growth, which helps treat both traveler’s diarrhea and irritable bowel syndrome with diarrhea. It effectively modulates gut microbiota and alleviates dysbiosis-related symptoms. Beyond antimicrobial activity, rifaximin modulates dysbiosis, strengthens the epithelial barrier, and exerts pregnane X receptor (PXR)-mediated anti-inflammatory and anti-angiogenic effects, with implications along the gut–liver and gut–brain axes. These pleiotropic properties support expanding uses in small intestinal bacterial overgrowth (SIBO), inflammatory bowel diseases (IBD), functional dyspepsia, bloating, and lactose intolerance. However, high cost and potential resistance with prolonged exposure highlight the need for antimicrobial stewardship. The aims of this review are to summarize licensed and emerging indications and delineate non-conventional mechanisms. Patient stratification and further controlled clinical studies are required to optimize rifaximin’s precision-medicine potential.

## Introduction

Rifaximin is a non-systemic, gut-selective antibiotic belonging to the rifamycin class, characterized by minimal absorption and high intraluminal bioavailability [1, 2]. Since its approval in 1987, it has become a cornerstone in the management of several gastrointestinal disorders due to its ability to act locally while modulating the intestinal microbiota. In this context, understanding its pharmacokinetic properties is essential to appreciate its clinical behavior. The antibiotic rifaximin belongs to the rifamycin class of broad-spectrum oral agents that target both Gram-positive and Gram-negative bacteria [1, 2]. As a semisynthetic derivative of rifamycin, it is expected to be absorbed in the gastrointestinal (GI) tract through passive diffusion. Nevertheless, due to its chemical structure, rifaximin remains ionized at all pH values along the GI tract [1]. This property results in extremely low systemic absorption, reported to be less than 0.4%, while enabling the drug to achieve high luminal concentrations throughout the GI tract [3]. Its high solubility in bile salts enhances its activity against both pathogenic and commensal bacterial flora of the small intestine, a bile-rich environment that ensures high levels of luminal bioavailability [4]. In contrast, within the colon, rifaximin is mainly concentrated in the aqueous luminal phase, where limited solubility due to its hydrophobic nature leads to reduced bioavailability, restricting its activity to highly susceptible bacterial species [4]. Rifaximin also shows limited absorption into the gut wall because its tendency to form polymeric aggregates hinders its passage across the mucosa. The rifaximin-α formulation maintains stability while being poorly soluble, thus enhancing its intestinal selectivity and minimizing systemic exposure. When taken orally, 97% of the drug is excreted unchanged in the feces, and stool concentrations reach 4,000–8,000 µg/g, well above the minimum inhibitory concentrations (MIC) for most enteropathogens [3]. Building on these pharmacokinetic properties, its pharmacodynamic profile further explains its therapeutic versatility. Mechanistically, rifaximin demonstrates an affinity for the β-subunit of bacterial DNA-dependent RNA polymerase (rpoB), through which it irreversibly blocks RNA synthesis and bacterial growth [5, 6]. Beyond its antimicrobial effect, exerts anti-inflammatory and barrier-protective properties. It has been shown to beneficially modulate gut microbiota composition, promoting commensal species such as *Faecalibacterium prausnitzii*, which down-regulates intestinal inflammation, while reducing *Clostridium* species [7]. In addition, activates the nuclear pregnane X receptor (PXR), leading to indirect inhibition of the nuclear factor κ-light-chain enhancer of activated B cells (NF-κB) pathway [1]. This mechanism contributes to the attenuation of intestinal inflammation and improvement of mucosal barrier integrity. In preclinical models, also prevented mucosal inflammation, barrier impairment, and visceral hyperalgesia in chronic stress paradigms such as water-avoidance and repeated restraint stress [8]. In patients with IBS, rifaximin also improved small-bowel permeability, accelerated colonic transit, and shifted fecal microbiome and organic-acid profiles, supporting a mechanistic link between barrier integrity, motility, and symptom relief [9]. Rifaximin exhibits ideal pharmacological features for treating GI disorders because it provides local antimicrobial and anti-inflammatory effects while avoiding systemic side effects typical of other antibiotics [10]. Consistent with these mechanistic insights, rifaximin is active against a broad range of enteric pathogens, including *Escherichia coli*, *Salmonella*, *Shigella*, *Campylobacter*, and *Clostridioides difficile*. Its approved indications include: Traveler’s diarrhea (200 mg three times daily for 3 days) [10]; hepatic encephalopathy (550 mg twice daily for recurrence prevention) [11]; Irritable bowel syndrome with diarrhea (IBS-D) (550 mg three times daily for 14 days) [12]. In addition to these established uses, rifaximin has been employed off-label in various conditions beyond its approved indications, and these emerging off-label uses and novel therapeutic perspectives will be discussed in detail in the following sections. Despite its broad-spectrum antimicrobial activity, rifaximin generally exhibits a low propensity for resistance development during short-term use; resistance, when it occurs, is typically linked to point mutations in rpoB, which are non-transferable between bacterial species [13, 14]. However, emerging evidence suggests that prolonged or repeated use may select for resistant strains. Turner et al. (2024, *Nature*) reported that long-term rifaximin prophylaxis in patients with liver disease selected *Enterococcus faecium* mutants carrying rpoB mutations conferring cross-resistance to daptomycin, a last-resort antibiotic [15]. As discussed by Artru and Hernández-Gea, these findings call for cautious and monitored long-term use to prevent selective pressure on the gut resistome while preserving rifaximin’s clinical benefits [16]. Similarly, Valentin et al. (2011) observed the emergence of rifampin-resistant *Staphylococcus* isolates following rifaximin exposure, suggesting a possible, though infrequent, cross-resistance within the rifamycin class [17]. Overall, rifaximin has a favorable safety profile, especially compared with systemically absorbed antibiotics. Due to minimal systemic exposure, most adverse events are mild and occur at rates comparable to placebo [18]. The most common include transient GI disturbances or upper respiratory symptoms (e.g., nausea, nasopharyngitis). Although uncommon, rifaximin’s broad antimicrobial spectrum may occasionally alter intestinal microbial balance and theoretically predispose to *Clostridioides difficile* overgrowth. Nevertheless, clinical data suggest this risk is very low. In a large retrospective study of cirrhotic patients receiving long-term rifaximin, the incidence of C. difficile infection (CDI) was significantly lower in the rifaximin group (12.8%) compared with non-rifaximin controls (29.7%) [19]. Isolated case reports have described rifaximin-associated CDI, particularly after repeated treatment cycles or in high-risk individuals [20].

This review aims to synthesize and contextualize the growing body of evidence that extends rifaximin’s therapeutic relevance beyond its approved gastrointestinal indications. By examining emerging off-label applications, it explores how rifaximin’s ability to modulate the intestinal environment, reduce inflammation, and influence host–microbiota interactions opens new perspectives in conditions traditionally considered outside the scope of non-systemic antibiotics. Particular emphasis is placed on innovative mechanistic insights, such as its impact on the gut–brain axis, barrier integrity, immune signaling, and angiogenic pathways, which collectively suggest a broader pharmacological potential than previously recognized. The review also intends to critically evaluate the robustness and limitations of current data, delineating areas where preliminary findings warrant further investigation.

## Host-directed mechanisms beyond antibacterial activity

### Anti-inflammatory properties via PXR activation

The key to rifaximin’s anti-inflammatory effect lies in its activation of the PXR. PXR functions as a xenobiotic sensor and regulator of genes involved in detoxification, but it also has profound effects on inflammation. PXR activation by rifaximin antagonizes NF-κB, resulting in a marked reduction in pro-inflammatory cytokine production. Studies have shown that rifaximin-treated intestinal cells or mice have significantly lower levels of tumor necrosis factor-α (TNF-α), interleukin-6 (IL-6), and interleukin-1β (IL-1β) in response to inflammatory stimuli [21, 22]. PXR activation also helps maintain tight-junction integrity. In mouse models of colitis, rifaximin significantly lessened disease severity only in mice with a humanized PXR gene. These humanized mice had less weight loss, improved colon histology, and reduced pro-inflammatory cytokine expression when given rifaximin compared to controls [23]. Rifaximin, via PXR, induces genes for antioxidant and anti-toxin responses, like CYP3A4 and multidrug resistance-1 (MDR1), which can mitigate cellular stress during inflammation [23, 24]. In a small trial on ulcerative colitis (UC) patients intolerant to steroids, 64% of those receiving adjunct rifaximin achieved clinical improvement with significant reductions in stool frequency, rectal bleeding, and sigmoidoscopic inflammation scores versus those on mesalamine alone [25]. Another open-label study reported a high remission rate when rifaximin was added to mesalamine maintenance therapy for a UC flare-up [26]. Patients with Crohn’s disease (CD) treated with rifaximin showed decreased mucosal TNF and IL-1β levels on biopsies [24]. Even in IBS, which was historically not considered an “inflammatory” condition, recent biopsies have found modest increases in gut immune cells and cytokines [27, 28]. Rifaximin’s ability to normalize these immune markers might partly explain the sustained symptom relief that some IBS-D patients experience after treatment.

### Anti-angiogenic effects

Chronic inflammation often couples with angiogenesis, perpetuating inflammation by increasing vascular permeability and leukocyte trafficking. Rifaximin has demonstrated anti-angiogenic effects in preclinical studies via PXR-dependent inhibition of angiogenic factors. Researchers have found that rifaximin can down-regulate vascular endothelial growth factor (VEGF), its receptor VEGFR2, and other pro-angiogenic mediators such as nitric oxide (NO) and matrix metalloproteinases (MMP-2 and MMP-9) [29].

In a study using Caco-2 cells and endothelial assays, rifaximin blocked multiple signaling pathways that drive angiogenesis such as reduced phosphorylation of ak transforming (Akt), mechanistic target of rapamycin (mTOR), and 70-kDa ribosomal protein S6 kinase (p70S6K), which are part of the nutrient-sensing pathway leading to hypoxia-inducible factor 1-α (HIF-1α) activation, and also the inhibition of the p38 mitogen-activated protein kinase (p38 MAPK)/NF-κB pathway [29]. Through these actions, rifaximin lowered HIF-1α levels and ultimately decreased the secretion of VEGF and NO from intestinal cells. When PXR was silenced, or absent, rifaximin no longer had the same impact on VEGF/NO or MMP expression. This indicates that rifaximin inhibition of angiogenesis is an extension of its PXR activation in intestinal and, perhaps, liver cells [30]. By decreasing MMP-2/9, rifaximin also impedes the breakdown of the extracellular matrix that normally allows endothelial cells to migrate and form new vessels. One outcome observed was reduced migration of Caco-2 cells in a wound-healing assay when treated with rifaximin [29].

Rifaximin could inhibit angiogenesis in small-bowel angiodysplasia by blocking an lncRNA (HIF1A-AS2)/microRNA-153/HIF-1α/Angiopoietin-2 axis that was discovered to drive angiogenesis in angiodysplastic lesions [31]. Was able to decrease Angiopoietin-2 by modulating this novel non-coding RNA pathway, thereby stunting new blood vessel formation in vitro [31].

In liver fibrosis, the angiogenesis is closely intertwined with fibrogenesis. The fibronectin produced by activated stellate cells acts as a scaffold for new blood vessels in the scar tissue. In a bile duct ligation mouse model, rifaximin-treated mice had significantly fewer hepatic microvessels and less expression of cluster of differentiation (CD31) than untreated fibrotic mice, correlating with reduced fibrosis [32].

### Intestinal hyperpermeability and abdominal pain

The term “leaky gut” has long been used to describe increased intestinal permeability, originally popularized through the pioneering work of Patrice Cani and colleagues in the mid-2000s [33]. However, in recent years, this concept has considerably expanded, with growing research exploring the potential roles of intestinal permeability in a wide range of conditions.

Through intestinal hyperpermeability, the epithelial barrier lets toxins, antigens, and bacteria enter the body, which sparks immune responses leading to body-wide inflammation. Rifaximin treatment has been associated with strengthening of the gut barrier and normalization of intestinal permeability, effects that appear to extend beyond its antimicrobial action. In experimental models, animals treated with rifaximin exhibited increased expression of tight-junctions, like zonula occludens-1 (ZO-1), occludin, and claudins, thereby preventing barrier disruption. In a mouse model of heat stroke, rifaximin-treated animals maintained gut integrity by up-regulating ZO-1/occludin and consequently reduced endotoxin levels and systemic inflammation [34]. Rifaximin treatment was also associated with enhanced mucin (MUC2) production via increased growth of short-chain fatty acids (SCFAs)-producing bacteria, such as *Lachnospiraceae* and *Ruminococcaceae*, which generate butyrate, further ameliorating the mucosal layer [34]. Rifaximin-treated rats exhibited higher *Lactobacillus* and *Lachnospiraceae* abundance and correspondingly normal levels of IL-6 and TNF-α in the gut, whereas untreated stressed rats displayed an inflammatory mucosa and barrier disruption [14]. In an observational cohort of symptomatic uncomplicated diverticular disease (SUDD), 6 months of cyclic rifaximin was associated with significant reductions in patients’ abdominal pain scores, and those improvements were linked to increases in *Akkermansia* and *Ruminococcaceae* in the fecal microbiome [35].

Rifaximin’s microbiota modulation may ease visceral hypersensitivity by reducing gas production and luminal irritants, decreasing the mechanical and chemical stimuli that activate visceral afferents. Animal models of visceral hyperalgesia have shown that rifaximin-treated animals exhibited higher pain thresholds and less neuronal activation in pain pathways than controls [14]. All the emerging effects of rifaximin are summarized in the following table [Table 1].

**Table 1:** Emerging effects of rifaximin: molecular pathways, biomarkers, and outcomes

Mechanism / Pathway	Molecular / Cellular Target	Experimental or Clinical Model	Main Outcomes	References
PXR activation and NF-κB inhibition	↑CYP3A4, ↑MDR1; ↓TNF-α, ↓IL-6, ↓IL-1β; NF-κB antagonism	Intestinal cell lines; humanized PXR mice; UC patients	↓Pro-inflammatory cytokines; improved mucosal integrity; clinical improvement in UC	[22, 23, 43]
Reinforcement of the intestinal barrier	↑ZO-1, ↑Occludin, ↑Claudins; ↑MUC2; ↑SCFAs (butyrate)	Mouse models of heat stress and chronic stress; SUDD cohort	Preserved tight junctions; ↓Endotoxemia; ↓Systemic inflammation and abdominal pain	[14, 34, 35]
Anti-angiogenic, PXR-dependent effects	↓VEGF/VEGFR2, ↓NO, ↓MMP-2/9; ↓p-Akt/mTOR/p70S6K; ↓p38 MAPK/NF-κB	Caco-2 and endothelial assays; PXR-silenced models; liver fibrosis mice	↓Angiogenic factor expression; ↓Cell migration; ↓Microvessel density (CD31)	[29–31]
Microbiota modulation	↑Faecalibacterium prausnitzii, ↑Bifidobacteria, ↑Akkermansia; ↓Clostridium/Enterobacteriaceae; ↑SCFAs	IBS-D, SUDD, IBD models	Restored eubiosis; ↓Mucosal inflammation; improved bowel symptoms	[7, 35, 50, 56]
Visceral hypersensitivity modulation	↓Neuronal activation in nociceptive circuits; ↑Visceral pain threshold	Animal models of visceral hyperalgesia	↓Visceral pain and inflammatory signaling	[14, 46, 49]
Gut–brain axis and neuroinflammation	Microglia anti-inflammatory shift; ↑Brain butyrate; ↓IL-6/TNF-α; restored connectivity (insula, hippocampus)	Stress/depression rat models; PD + SIBO patients; HE imaging studies	↓Depressive-like behaviors; improved motor/cognitive performance; normalized brain connectivity	[74–83]
Gut–liver axis and TLR4 modulation	↓TLR4 signaling; improved epithelial barrier	Liver fibrosis models; HE patients	↓Fibrogenesis and angiogenesis; ↓HE recurrence; gut–liver axis protection	[31, 54–56]

Abbreviations: PXR, pregnane X receptor; NF-κB, nuclear factor κ-light-chain enhancer of activated B cells; CYP3A4, cytochrome P450 3A4; MDR1, multidrug resistance protein 1; TNF-α, tumor necrosis factor-α; IL-6, interleukin-6; IL-1β, interleukin-1β; ZO-1, Zonula occludens-1; MUC2, mucin 2; SCFAs, short-chain fatty acids; VEGF, vascular endothelial growth factor; VEGFR2, vascular endothelial growth factor receptor 2; NO, nitric oxide; MMP-2/9, matrix metalloproteinases 2 and 9; Akt, protein kinase B; mTOR, mechanistic target of rapamycin; p70S6K, 70-kDa ribosomal protein S6 kinase; MAPK, mitogen-activated protein kinase; TLR4, toll-like receptor 4; UC, ulcerative colitis; IBD, inflammatory bowel disease; IBS-D, irritable bowel syndrome with diarrhea; SUDD, symptomatic uncomplicated diverticular disease; PD, parkinson’s disease; SIBO, small intestinal bacterial overgrowth; HE, hepatic encephalopathy. Arrow symbols indicate direction of change: ↑ increase or upregulation; ↓ decrease or downregulation

## Licensed therapeutic applications

### Traveler’s diarrhea (TD)

TD represents a frequent illness that affects people visiting areas with inadequate sanitation systems primarily through enterotoxigenic or enteroaggregative *Escherichia coli* (ETEC and EAEC) [36], but also by other bacterial or viral pathogens. TD symptoms include diarrhea, abdominal cramps, nausea, and sometimes fever or vomiting, and, in severe cases, dehydration [37, 38]. Standard treatment involves rehydration and antibiotics such as fluoroquinolones or trimethoprim-sulfamethoxazole. Systemic antibiotics can disrupt the native gut flora and carry risks like antibiotic-associated diarrhea or resistance [39]. Rifaximin serves as a safer and targeted alternative for TD thanks to its local therapeutic action in the GI tract without significant systemic absorption [40].

Randomized trials showed that short rifaximin courses (200–400 mg t.i.d. for 3 days) are as effective as fluoroquinolones, shortening illness duration and achieving ~ 80–90% cure rates in non-invasive TD [41, 42]. Notably, rifaximin is only indicated for TD caused by non-invasive *Escherichia coli*, it should not be used if invasive pathogens like *Campylobacter* or *Shigella* are suspected. Fortunately, most TD (> 80%) is caused by non-invasive organisms such as ETEC and EAEC [43].

Prophylactic use (200–600 mg/day) reduced TD incidence by ~ 60–70% in travelers to endemic regions [44, 45]. Given concerns about promoting resistance, routine chemoprophylaxis for all travelers is not generally recommended; however, it may be considered in high-risk individuals such as those with inflammatory bowel disease (IBD) or immunosuppression traveling to high-TD regions [44]. Rifaximin had minimal impact on the overall resistome and has not been associated with significant resistance development in enteric flora in these short-term uses [14].

### Irritable bowel syndrome with diarrhea (IBS-D)

IBS is a functional GI disorder characterized by recurrent abdominal pain associated with altered bowel habits. In IBS-D, patients experience loose stools, urgency, and bloating. Growing evidence implicates alterations of the gut microbiota and low-grade mucosal inflammation in IBS-D pathogenesis, supporting the therapeutic rationale for non-absorbable antibiotics such as rifaximin [46]. In particular, small intestinal bacterial overgrowth (SIBO) or other dysbiosis has been proposed as a contributor in some IBS patients. The association between SIBO and the pathogenesis of IBS-D has been strengthened by evidence of gut mucosal inflammation, a higher prevalence of SIBO in these patients, compared with controls [47]. Rifaximin (550 mg t.i.d. for 14 days) demonstrated significant benefit in two phase III trials (TARGET 1 & 2), improving global IBS symptoms compared with placebo, with notable relief of bloating, abdominal pain, and stool consistency [12]. The TARGET 3 randomized trial demonstrated that repeat treatment with rifaximin is safe and effective in IBS-D patients [18]. Consistently, a subsequent analysis of stool microbial antibiotic sensitivity from the same trial cohort showed no clinically significant changes in antimicrobial susceptibility following repeat treatment [48]. Recent analyses using composite endpoints showed superior simultaneous improvement in abdominal pain, bloating, and urgency with rifaximin [49]. Clinical benefit typically appears within one week and may persist for several weeks post-therapy; up to two retreatments maintain efficacy and safety [41]. In a study conducted in CD patients, rifaximin was shown to enhance beneficial taxa such as *Faecalibacterium prausnitzii* and Bifidobacteria, supporting its eubiotic potential [50] In IBS-D patients, rifaximin treatment was associated with increased SCFAs production and improved intestinal permeability, contributing to reduced visceral hypersensitivity [46]. Consistently, a mechanistic clinical study showed normalization of intestinal permeability, faster colonic transit, and microbiome/organic-acid modulation after rifaximin in IBS [9]. The overall result is normobiotic conditions that alleviate IBS-D symptoms with minimal systemic absorption [49]. Recent studies suggest that specific microbial and metabolic profiles may predict rifaximin responsiveness, patients enriched in *Bifidobacterium* and depleted in *E. coli* and *Enterobacter* appear to benefit most [51]. Ongoing research integrating microbiome and metabolomic data aims to refine patient selection and optimize sustained remission [52].

The therapeutic benefit of rifaximin, however, tends to wane over time. In the TARGET 3 randomized trial, among initial responders, 64.4% experienced symptom relapse within 18 weeks of follow-up. A repeat two-week course of rifaximin achieved a clinical response in 38.1% of relapsing patients versus 31.5% on placebo [18]. These findings highlight that rifaximin provides symptom relief rather than a cure, and that its efficacy may decrease with repeated courses [12].

### Hepatic encephalopathy (HE)

HE is a neuropsychiatric complication of chronic liver disease resulting from the inability to detoxify gut-derived neurotoxins like ammonia by the liver. Accumulation of ammonia and other substances affects the brain, leading to confusion, altered level of consciousness, and if untreated, coma. Rifaximin has become a key therapy in both acute and recurrent HE because it targets ammonia-producing gut bacteria. In the pivotal randomized trial by Bass et al. (2010), rifaximin 550 mg b.i.d. for 6 months significantly reduced HE recurrence-related hospitalizations, improving cognition and quality of life in cirrhotic patients [11]. Combination with lactulose, which acts by trapping ammonia in the colon, provides synergistic benefit and is now standard for secondary prophylaxis. In acute HE, adjunctive rifaximin accelerated mental status recovery and increased reversal rates compared with lactulose alone [53]. Beyond ammonia reduction, rifaximin beneficially modulates the gut–liver axis [54] and sustains epithelial health [55, 56]. Experimental models indicate antifibrotic and antiangiogenic actions via toll-like receptor 4 (TLR4) pathway modulation [32]. Clinically, long-term therapy has correlated with improved hepatic function indices, possibly secondary to reduced HE episodes [57].

In a subset of cirrhotic patients presenting a Parkinson-like HE phenotype, linked to portosystemic shunting and manganese deposition, rifaximin has shown unexpected benefits. Case reports describe rapid improvement of extrapyramidal symptoms, including rigidity, tremor, and gait freezing, after 4 weeks of rifaximin 600 mg b.i.d., even when lactulose had failed [58–61]. These findings suggest that by lowering gut-derived neurotoxins and systemic inflammation, rifaximin may relieve both cognitive and motor features of HE, reinforcing its central role in the gut–liver–brain therapeutic continuum.

## Emerging off-label clinical applications

A growing body of preclinical and early clinical research is now investigating the potential of rifaximin in a variety of gut-related and systemic conditions. Although these studies remain relatively limited and heterogeneous, they offer important and pioneering insights into the broader pharmacological profile of rifaximin, extending beyond its traditional antimicrobial activity.

### Small intestinal bacterial overgrowth (SIBO)

SIBO is a condition in which excessive bacteria colonize the small intestine, leading to fermentation, gas production, and digestive symptoms such as bloating, flatulence, abdominal pain, and diarrhea [13]. It is frequently associated with functional GI disorders, particularly IBS, or with anatomical abnormalities of the gut. SIBO can present as hydrogen-, hydrogen sulfide–, or methane-predominant (intestinal methanogen overgrowth, IMO), reflecting distinct microbial ecosystems and metabolic pathways. Hydrogen- and hydrogen sulfide-dominant forms, often linked to IBS-D, are associated with reduced microbial diversity and overgrowth of sulfate-reducing bacteria such as *Fusobacterium* and *Desulfovibrio spp.*, which contribute to diarrhea and abdominal discomfort. Conversely, methane-predominant SIBO/IMO, associated with *Methanobrevibacter smithii*, *Methanosphaera stadtmanae*, and *Methanomassiliicoccus luminyensis*, correlates with constipation through methane-mediated slowing of intestinal transit. These gas-specific profiles illustrate how microbial metabolism can directly influence motility and symptom patterns [62]. Rifaximin is considered a first-line antibiotic for SIBO due to its local action and broad spectrum against gut microbes [63].

A systematic review and meta-analysis by Gatta and Scarpignato (2017), encompassing 32 studies (1331 patients), reported an overall SIBO eradication rate of approximately 70% with rifaximin (1200–1600 mg/day) and symptomatic improvement in 68% of cases, with only 4.6% experiencing mild GI side effects [5]. A Chinese study showed that a 2-week course of rifaximin normalized lactulose breath tests in nearly half of SIBO-positive IBS-D patients, significantly improving bloating, pain, and stool urgency for up to 10 weeks after treatment [46]. These findings indicate that rifaximin may induce a sustained “microbial reset,” restoring normobiotic conditions and alleviating visceral hypersensitivity and hypermotility.

Not all patients respond to a single course; however, repeat treatment has proven effective in partial responders, further reducing breath-test gas levels and symptoms [64]. In methane-predominant forms, combination therapy with neomycin has achieved higher eradication rates than rifaximin alone [64]. Rifaximin selectively targets overgrown or pathogenic bacteria while sparing beneficial commensals and promoting recolonization by *Lactobacillus* and *Bifidobacterium* species [56]. These genera produce lactic acid and SCFAs, which nourish enterocytes, reinforce the mucosal barrier, and suppress opportunistic pathogens.

### Inflammatory bowel disease (IBD)

Beyond SIBO, rifaximin has been investigated in IBD characterized by microbial dysbiosis and mucosal encroachment. In mild-to-moderate CD, open-label studies reported remission rates up to ~ 69%, and a controlled trial with rifaximin-extended intestinal release showed higher (though not statistically significant) remission rates than placebo after 12 weeks [65]. In UC, adjunctive rifaximin (400 mg t.i.d. for 4 weeks) improved remission rates when added to mesalamine [26]. Adding rifaximin to standard mesalamine therapy in mild UC might improve remission rates, as small studies hint [65–67]. Mechanistically, rifaximin increases *Faecalibacterium prausnitzii*, a butyrate-producing species inversely correlated with CD flares, and decreases aggressive *Enterobacteriaceae*, thereby strengthening the mucosal barrier and promoting eubiosis [56]. In CD, rifaximin could be used in post-operative recurrence prevention or to treat mild flares, capitalizing on its gut-directed action without immunosuppression [65]. Large and well-controlled trials are needed to define its role, optimal dose, and treatment duration in IBD.

### Functional dyspepsia (FD)

Emerging evidence suggests that rifaximin can alleviate symptoms in FD, likely by targeting gut dysbiosis. In a randomized controlled trial, a 2-week course of rifaximin (1200 mg/day) provided significantly greater relief of FD symptoms compared to placebo [68]. By 8 weeks after treatment, 78% of rifaximin-treated patients reported adequate relief of global dyspeptic symptoms versus 52% on placebo [69]. Rifaximin was particularly effective for belching and post-prandial fullness/bloating. At week 4, only 20% of rifaximin patients had moderate-to-severe bloating compared to 43% of placebo patients. Notably, female patients appeared to derive the most benefit in symptom improvement. Further supporting this, in a comparative study examining the effects of rifaximin in FD patients with or without concomitant IBS, treatment with rifaximin led to a significant improvement in GI symptoms and visceral sensory function, with a statistically significant (> 25%) reduction in the structured assessment of GI symptoms (SAGIS) dyspepsia and the diarrhea subscores, and this response was maintained at the 6-week assessment [69].

### Functional bloating and abdominal distension

For patients suffering from chronic bloating and excessive gas, rifaximin has demonstrated efficacy in reducing these symptoms. An early randomized trial in 126 adults with at least 12 weeks of bloating and flatulence showed that a 10-day treatment with rifaximin (800 mg/day) led to significantly symptom improvement than placebo [70]. By the end of treatment, 41% of patients on rifaximin reported their bloating/gas symptoms improved, versus 23% on placebo, and this benefit persisted 10 days post-treatment (29% vs. 12%) [70]. The benefit was observed in patients both with and without IBS. These results support the idea that abnormal fermentation by gut bacteria contributes to functional bloating and that targeting the gut microbiome with a non-absorbed antibiotic can provide relief. Additionally, in a study focusing on FGIDs (functional GI disorders), positive effects on symptom improvement were observed: results showed a significant reduction in abdominal bloating and distension with rifaximin compared with placebo [71].

### Enhanced lactase activity and lactose intolerance

Another intriguing effect of rifaximin is its potential to improve lactose intolerance by increasing lactase enzyme activity. SIBO is known to impair digestive enzymes like lactase, contributing to lactose malabsorption [72]. A recent pilot study in IBS-D patients with co-existing SIBO and lactose intolerance demonstrated that 2 weeks of rifaximin markedly enhanced lactase function, the proportion of patients with abnormal lactose breath tests dropped from 88% to 52% after rifaximin, and a urinary D-xylose absorption test showed a significant increase in lactase activity [72]. Alongside 60% of patients reported improvement in abdominal pain, and nearly half reported less bloating. Earlier clinical work also supports this evidence, 10 days of rifaximin significantly reduced breath hydrogen production and symptom scores in lactose-intolerant patients, an effect comparable to a 6-week lactose-free diet [73].

## New opportunities and therapeutic directions

### From gut wellness to brain wellness: the gut-brain axis

The gut-brain axis is a hot topic, and rifaximin effects on this axis are being unraveled. Depression and anxiety have been linked to gut dysbiosis and inflammation. A fascinating study in rats showed that rifaximin prevented the development of depression-like behaviors induced by chronic stress by modulating microglial activation in the brain and increasing brain levels of butyrate [74]. Rifaximin treatment was associated with higher abundance of *Ruminococcus bromii* and *Lachnospiraceae* in the gut, which are major butyrate producers, and indeed, brain butyrate was elevated in rifaximin-treated rats [74]. The rats also had reduced neuroinflammation as indicated by less activated microglia and normalized levels of IL-6 and TNF-α in brain tissue. Rifaximin also increased anti-inflammatory factors from microglia and normalized hippocampal neurogenesis that was impaired by stress [74]. Li et al. demonstrated that rifaximin could protect adolescent rats from developing depressive behaviors by this gut microbiota-microglia mechanism [74]. These findings suggest a possible therapeutic angle for rifaximin in depression and perhaps other neuroinflammatory conditions. It is noteworthy that butyrate itself has antidepressant effects [75], and rifaximin’s ability to boost butyrate-producing gut flora might thus indirectly confer mood benefits. Parkinson’s disease (PD) has emerged as another potential target for rifaximin gut-mediated effects. PD patients commonly exhibit intestinal dysbiosis and SIBO, which is reported in up to ~ 50% of cases, linked to worse motor fluctuations and increased gut inflammation [76, 77]. In a transgenic PD mouse model, long-term rifaximin treatment shifted the microbiota and markedly reduced systemic inflammatory cytokines, and preserved blood-brain barrier integrity. Treated PD mice showed improved motor performance and memory with lower microglial activation and greater neuronal survival compared to controls [78]. A small open-label trial in PD patients with co-existing SIBO found that a 7-day course of rifaximin (1200 mg/day) eradicated SIBO in nearly 80% of patients and significantly improved motor “off” time and delayed-on phenomena, leading to smoother motor responses [76]. No major changes in levodopa levels were observed, suggesting the benefit was due to improved gut conditions, and about 43% of patients had SIBO relapse by 6 months post-treatment [76]. These findings highlight that rifaximin might positively influence the gut-brain axis in PD, not only easing GI symptoms and enhancing drug absorption but possibly attenuating neuroinflammation and disease progression. Given its safety and tolerability, further controlled trials are warranted to explore rifaximin as an adjunct therapy in PD by targeting gut dysbiosis as a means to modify the disease course.

In cirrhotic patients with HE, rifaximin reduces gut-derived neurotoxins and inflammation, leading to cognitive gains. Cirrhotics with minimal HE treated with rifaximin show significantly improved psychometric test performance and quality-of-life scores, along with a sharp drop in plasma endotoxin [79, 80]. Rifaximin-treated patients exhibit increased activation in subcortical circuits (thalamus, caudate, insula, hippocampus) during working-memory tasks, and stronger connectivity of executive/motor networks [79]. These brain changes correlated with lower blood ammonia and endotoxin. In HE, the gut-targeted antibiotic led to improved cognitive flexibility, attention, and memory, effectively “cleaning up” the gut so the brain could work better [79]. Furthermore, rifaximin led to a global reorganization of brain networks. Responders showed increased thalamic and insular connectivity and needed less frontal activation to perform cognitive tasks. Rats with mild liver damage develop hippocampal neuroinflammation, high TNF-α, microglial activation, and impaired spatial memory. Daily rifaximin reversed these effects and normalized inflammatory cytokines, glutamate receptor expression in the hippocampus, and rescued learning and memory [81]. In adolescent rats exposed to chronic unpredictable mild stress, rifaximin preserved brain health and prevented depressive/anxiety behaviors, increased gut bacteria that produce butyrate, and raised brain butyrate levels. This shifted microglia toward an anti-inflammatory state, reducing neuroinflammation [74]. Another study found rifaximin regulated tryptophan metabolism in stressed rats, lowering the “toxic” kynurenine pathway and boosting serotonin turnover in the hippocampus, and thereby improved depression-like behavior [82]. In SIBO patients treated with rifaximin, urinary metabolomics showed lower kynurenine and quinolinic acid and higher 5-hydroxyindoleacetic acid/tryptophan ratios, reflecting greater serotonin turnover [83]. Taken together, these proof-of-concept studies show that by rebalancing the microbiome and its metabolites, rifaximin can limit systemic inflammation and keep brain immune cells calm, preventing the anhedonia and cognitive slowing seen in neuroinflammation [82]. In healthy volunteers, 7-day rifaximin treatment produced widespread changes in resting-state brain connectivity, especially involving the insular cortex, a key interoceptive hub. In that study, all the connectivity clusters affected by rifaximin included the insula, suggesting that gut microbiota changes were rapidly communicated to the brain [80]. Notably, brain imaging after rifaximin shows that patients who improve in gut symptoms tend to have post-treatment connectivity patterns resembling healthy controls, implying a “reset” of dysregulated gut-brain signaling [80]. The information discussed in the previous section is summarized in Table 2, which provides an overview of the key findings and concepts presented above.

**Table 2:** Approved and emerging off-label therapeutic applications of rifaximin

Indication	Approval status	Typical regimen	Key clinical findings/outcomes	References
Traveler’s diarrhea (non-invasive *E. coli*)	Approved	200 mg TID for 3 days	Comparable efficacy to fluoroquinolones; cure rate ≈ 80–90%; prophylaxis reduces TD incidence by 60–70%	[10, 40–45]
Irritable bowel syndrome with diarrhea (IBS-D)	Approved	550 mg TID for 14 days; up to two retreatments	TARGET 1–2: significant global symptom improvement (pain, bloating, stool consistency); TARGET 3: retreatment response 38% vs. 31.5% placebo	[12, 18, 49]
Hepatic encephalopathy (HE)	Approved	550 mg BID (chronic use), often + lactulose	↓HE recurrence and hospitalizations; improved cognition and QoL; synergistic effect with lactulose	[11], 53– [57, 79]
Small-intestinal bacterial overgrowth / intestinal methanogen overgrowth (SIBO / IMO)	Off-label	1200–1600 mg/day for 10–14 days; + neomycin for methane-predominant type	Meta-analysis: ~70% eradication; 68% symptom improvement; higher efficacy in methane type when combined with neomycin	[5, 46, 64]
Inflammatory bowel disease (UC / CD, mild–moderate)	Off-label	UC: 400 mg TID for 4 weeks; CD: extended-release formulations	Adjunct to mesalamine improves remission rates and reduces mucosal cytokines (TNF-α, IL-1β)	[26, 65–67]
Functional dyspepsia (FD)	Off-label	1200 mg/day for 2 weeks	RCT: superior global symptom relief; marked benefit for post-prandial fullness and bloating persisting ≥ 8 weeks	[68, 69]
Functional bloating / abdominal distension	Off-label	800 mg/day for 10 days	RCT: significant improvement in bloating/flatulence vs. placebo; sustained effect 10 days post-treatment	[70, 71]
Lactose intolerance (with SIBO)	Off-label	1200 mg/day for 10–14 days	↓Abnormal lactose breath tests (88% → 52%); ↓abdominal pain and bloating; effect comparable to 6-week lactose-free diet	[72, 73]
Parkinson’s disease (with SIBO)	Off-label	1200 mg/day for 7 days	~ 80% SIBO eradication; ↓motor fluctuations and “off” time; improved cognition and neuroinflammation markers	[76, 78]
Parkinsonian phenotype of hepatic encephalopathy	Approved (sub-group of HE)	600 mg BID for ≈ 4 weeks	Rapid improvement in rigidity, tremor, and gait freezing in lactulose-non-responders	[58–61]

Abbreviations: TD, traveler’s diarrhea; IBS-D, irritable bowel syndrome with diarrhea; HE, hepatic encephalopathy; SIBO, small intestinal bacterial overgrowth; IMO, intestinal methanogen overgrowth; UC, ulcerative colitis; CD, crohn’s disease; FD, functional dyspepsia; QoL, quality of life; RCT, randomized controlled trial; TID, three times daily; BID, twice daily. Arrow symbols indicate direction of change: ↓ decrease or reduction

## Perspectives and conclusions

**Fig. 1:**
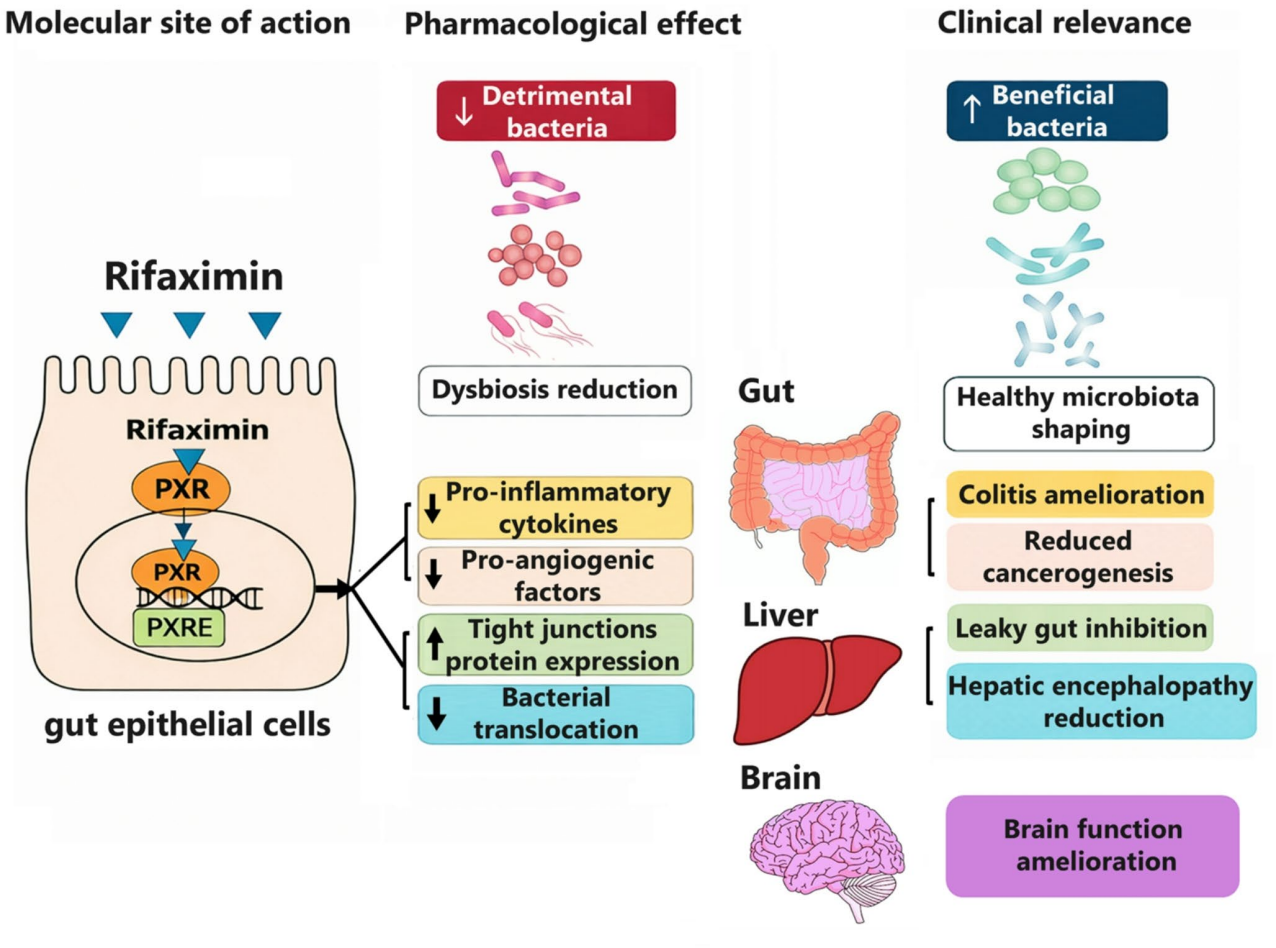
Molecular mechanism, pharmacological effects, and clinical relevance of rifaximin. [1, 14, 22–24, 29, 31, 32, 34, 54–56, 74, 78–81]. Abbreviations: PXR, pregnane X receptor. Arrow symbols indicate direction of change: ↑ increase or upregulation; ↓ decrease or downregulation. This figure was generated in part using https://BioRender.com

Rifaximin has proven to be a multifaceted therapeutic agent, earning its place as a “classic” antibiotic with continuously expanding uses. Its unique pharmacological profile, virtually non-absorbed, gut-focused, broad-spectrum yet microbiota-sparing, allows it to function dually as an antimicrobial and as a modulator of the host-microbiome interaction. The evidence reviewed here highlights several key points and future directions (Fig. 1), such as GI health, liver disease, and anti-inflammatory and anti-angiogenic roles, but also the gut-brain axis.

In an era where microbial triggers are suspected in many inflammatory diseases, rifaximin might serve as a template for new interventions targeting those triggers while simultaneously reinforcing barrier function. An important future direction lies in personalized therapy. Not all patients respond equally to rifaximin, and recent work has started to identify predictors of response. Rezaie et al. demonstrated that IBS-D patients with an abnormal baseline breath test were significantly more likely to respond to rifaximin, and that normalization of breath-test patterns predicted sustained clinical remission [84]. Complementary studies by Villanueva-Millán et al. have characterized distinct microbiome and gas-production signatures, hydrogen/hydrogen sulfide profiles in IBS-D and methane in IBS-C, suggesting that microbial composition may guide therapeutic selection and retreatment strategies [62]. Incorporating these biomarkers into practice could refine patient selection and enhance long-term outcomes.

Despite its favorable safety profile, caution is warranted regarding antimicrobial resistance. While rifaximin was long considered at low risk because of its minimal systemic absorption, Turner et al. reported that prolonged prophylaxis in cirrhotic patients selected *Enterococcus faecium* mutants carrying *rpoB* mutations conferring cross-resistance to daptomycin, a critical last-line antibiotic [15]. This observation challenges the notion that locally acting antibiotics are exempt from selective pressure and underscores the importance of antimicrobial stewardship and surveillance, particularly with chronic or repeated use.

Finally, the extension of rifaximin’s benefits to the gut–brain axis remains an area of active investigation. The concept that a non-absorbed antibiotic might influence mood or cognition through microbiome modulation is a powerful example of systems medicine and opens avenues for future microbiota-targeted neurotherapeutics.

In conclusion, rifaximin exemplifies how a well-established antibiotic can evolve into a precision-medicine tool addressing both infection- and inflammation-driven disorders. However, most emerging indications are still supported by small or open-label studies; robust, controlled trials are essential to confirm these effects. By combining evidence-based practice, personalized approaches, and prudent use, rifaximin’s therapeutic potential can be fully realized while preserving its long-term safety and efficacy.

## Data Availability

Data sharing is not applicable to this article as no new data were created or analyzed in this study.

## Abbreviations

AKT: Ak Transforming
CaCo: 2-Cancer Colon 2
CD: Crohn’s Disease
CD3: Cluster of Differentiation
EAEC: Enteroaggregative Escherichia coli
ETEC: Enterotoxigenic Escherichia coli
FD: Functional Dyspepsia
FGIDs: Functional Gastrointestinal Disorders
FODMAP: Fermentable Oligosaccharides, Disaccharides, Monosaccharides and Polyols
GI: Gastrointestinal
HE: Hepatic Encephalopathy
HIF: 1α-Hypoxia-Inducible Factor 1α
IBD: Inflammatory Bowel Disease
IBS: Irritable Bowel Syndrome
IBS: C-Irritable Bowel Syndrome Constipation Predominant
IL: 6-Interleukin-6
IMO: Intestinal Methanogen Overgrowth
MDR1: Multidrug Resistance 1
MIC: Minimum Inhibitory Concentration
MMP: 2-Matrix Metalloproteinase-2
MMP: 9-Matrix Metalloproteinase-9
mTOR: Mechanistic Target of Rapamycin
MUC2: Mucin 2
NF: kB-Nuclear Factorκ-Light-Chain Enhancer of Activated B Cells
NO: Nitric Oxide
PD: Parkinson’s Disease
PXR: Pregnane X Receptor
P38MAPK: p38 Mitogen-Activated Protein Kinase
p70S6K − 70: kDa Ribosomal Protein S6 Kinase
rpoB: Bacterial DNA-Dependent RNA Polymerase
SAGIS: Structured Assessment of Gastrointestinal Symptoms
SCFAs: Short-Chain Fatty Acids
SIBO: Small Intestinal Bacterial Overgrowth
SUDD: Symptomatic Uncomplicated Diverticular Disease
TBS: D-Irritable Bowel Syndrome With Diarrhea
TD: Traveler’s Diarrhea
TLUS: Time To Last Unformed Stool
TLR4: Toll-Like Receptor 4
TNFα: Tumor Necrosis Factor-α
UC: Ulcerative Colitis
UTIs: Urinary Tract Infections
VEGF: Vascular Endothelial Growth Factor
VEGFR2: Vascular Endothelial Growth Factor Receptor 2
VREfm: Vancomycin-Resistant Enterococcus Faecium
ZO-1: *Zonula Occludens-1*

## Declarations

### The use of artificial intelligence (AI) tools

The authors declare that no AI – based tools were used in the preparation, writing, or editing of this manuscript.
